# The association between preoperative 25-OH vitamin D levels and postoperative complications in patients undergoing colorectal cancer surgery

**DOI:** 10.1186/s12893-021-01369-y

**Published:** 2021-10-19

**Authors:** B. Balci, G. Kilinc, B. Calik, C. Aydin

**Affiliations:** 1 Department of General Surgery, Ankara Oncology Training and Research Hospital, Ankara, Turkey; 2 Department of General Surgery, Tepecik Training and Research Hospital, Izmir, Turkey

**Keywords:** Colorectal cancer, 25-OH vitamin D, Infectious complications, Surgical site infection

## Abstract

**Background:**

Determining the modifiable risk factors for postoperative complications is particularly significant in patients undergoing colorectal surgery since those are associated with worse long-term outcomes.

**Methods:**

Consecutive newly diagnosed 104 colorectal cancer patients were prospectively included in this single-center observational study. Preoperative serum 25-OH vitamin D levels were measured and analyzed for infectious and postoperative complications.

**Results:**

Serum 25-OH vitamin D levels were found to be < 20 ng/ml in 74 patients (71.2%) and ≥ 20 ng/ml in 30 patients (28.8%); and the mean serum 25-OH vitamin D level was 15.95 (± 9.08) ng/ml. In patients with surgical site infection and infectious complications, 25-OH vitamin D levels were significantly lower than patients without complications (p = 0.036 and p = 0.026). However, no significant difference was demonstrated in 25-OH vitamin D levels according to overall postoperative complications.

**Conclusions:**

Our results suggest that vitamin D levels might be a potential risk factor for infectious complications in patients undergoing colorectal cancer surgery.

## Background

Colorectal cancer (CRC) is the second cause of cancer-related mortality worldwide, and radical resection is still the treatment of choice [1]. Despite improvements in postoperative care, the rate of postoperative complication (PoC) is changed between 19 and 30% in patients undergoing elective colorectal surgery [2]. The most known factors related to PoC are nutritional status, comorbid diseases, and operation timing [3–5]. Inflammatory markers such as C-reactive protein (CRP), albumin, and procalcitonin have been widely used in clinical practice for early detection of complications [6–9]. As reported by numerous studies, PoC has a significant impact on not only short-term outcomes but also on overall survival in colorectal cancer patients [10–12].

25-hydroxy (OH) vitamin D levels have been inversely related to colorectal cancer risk by causing adenoma formation in colon epithelium [13–16]. 25-OH vitamin D levels have also been associated with long-term survival and increased response to oncological treatment; however, its effect on PoC has not been thoroughly investigated [17, 18].

The present study aimed to evaluate the association between serum 25-OH vitamin D levels and PoC and observe its effect on short-term outcomes in colorectal cancer patients.

## Methods

This study was designed as an observational cohort study; and was approved by the Ethics Committee of Izmir Tepecik Training and Research Hospital, University of Health Sciences, Turkey. The study protocol is performed in accordance with the relevant guidelines. All patients' written informed consents were taken after oral and written explanations were made.

### Patients' selection

We performed this study in patients undergoing colorectal cancer surgery between September 2017 and March 2019 at the department of General Surgery, Izmir Tepecik Training and Research Hospital, University of Health Sciences, Turkey. Consecutive patients who met the inclusion criteria were prospectively included in this study. The inclusion criteria were as follows: (1) histopathological evidence of colorectal cancer, (2) patients older than 18 years, (3) patients who underwent curative resection. The exclusion criteria were as follows: (1) unresectable disease, (2) systemic metastases, (3) patients who received neoadjuvant chemoradiotherapy, (4) patients who underwent an emergent operation or operated with palliative intent.

### Data collection

The routine preoperative workup, including complete blood count, liver and kidney function tests, were done with an addition of preoperative serum levels of 25-OH vitamin D and carcinoembryonic antigen (CEA). Blood samples were taken in a week to the scheduled operation in all patients. Patients' nutritional status was evaluated using nutritional risk score-2002 (NRS), and comorbid diseases were classified according to the American Society of Anesthesiologists (ASA) [19, 20]. Patients' operative data were noted, and PoC occurring within 30 days of surgery were graded based on Clavien-Dindo classification [21]. Pathology results were also recorded, including histopathological grading and pathological tumor-node-metastasis (pTNM) staging [22].

Preoperative 25-OH vitamin D levels were evaluated according to the guidelines of the American Endocrine Society [23]: severe deficiency (< 10 ng/ml), mild deficiency (10–20 ng/ml), level of insufficiency (20–30 ng/ml) and optimal levels (> 30 ng/ml).

### Study outcomes

Our study's primary outcome was to demonstrate the relation between 25-OH vitamin D levels and postoperative complications. The secondary outcome was to evaluate its relationship with histopathological grading and pTNM staging.

### Statistical analysis

The data was recorded and analyzed with IBM SPSS Statistics 24.0.0.0. Categorical variables were presented as frequency distributions and continuous variables as mean values ± standard deviation (median; min–max). Shapiro–Wilk test was performed to confirm the normal distribution of continuous variables. Categorical variables were analyzed using Chi-square and Fischer's exact tests. The Student t-test or Mann–Whitney-U test were used for analyses involving binary categorical subgroups, and One-way ANOVA and Kruskal–Wallis tests for analyses involving three or more categorical subgroups.

Logistic regression analysis in backward stepwise (likelihood ratio) fashion was performed to demonstrate independent risk factors for infectious complications. Selected co-variates were, including gender, age, ASA, NRS, serum levels of albumin, CEA, 25-OH vitamin D and WBC, type of procedure, T and N stages, and histopathological grade.

p value < 0.05 was accepted as significant for each test.

## Results

The study population consisted of 104 patients; 64 males (61.5%) and 40 females (38.5%); the mean age was found to be 62.71 (± 12.41) years. The mean serum 25-OH vitamin D level was 15.95 (± 9.08) ng/ml, which corresponds to the cohort's vitamin D deficiency. Serum vitamin D levels were found to be < 20 ng/ml in 74 patients (71.2%) and ≥ 20 ng/ml in 30 patients (28.8%). Patients' demographic and clinical characteristics are shown in Table 1.

**Table 1:** Patients' demographic and clinical characteristics stratified according to serum 25-OH vitamin D levels

Variables	Study population N (%) or mean (± SD) (n = 104)	25-OH Vit D < 20 ng/ml N (%) or mean (± SD) (n = 74)	25-OH Vit D ≥ 20 ng/ml N (%) or mean (± SD) (n = 30)	p value
Age (years)	62.71 (± 12.41)	63.28 (± 11.76)	61.30 (± 14.01)	0.463
Gender:				0.516
Female	40 (38.5%)	27 (36.5%)	13 (43.4%)	
Male	64 (61.5%)	47 (63.5%)	17 (56.7%)	
ASA scores:				0.043*
1	17 (16.3%)	8 (10.8%)	9 (30%)	
2	56 (53.8%)	41 (55.4%)	15 (50%)	
3	31 (29.8%)	25 (33.8%)	6 (20%)	
NRS scores:				0.106
0	58 (55.8%)	38 (51.4%)	20 (66.7%)	
1	31 (29.8%)	22 (29.7%)	9 (30%)	
2	15 (14.4%)	14 (18.9%)	1 (3.3%)	
CEA (µg/l)	7.4 (± 11.6)	7.44 (± 12.94)	6.56 (± 6.76)	0.758
Albumin (gr/dl)	3.8 (± 0.5)	3.88 (± 0.51)	3.87 (± 0.49)	0.832
Hemoglobin (gr/l)	12.6 (± 1.99)	12.11 (± 2.0)	12.26 (± 1.98)	0.714
WBC (10^3^/µl)	8080 (± 2274)	8340 (± 2479)	7440 (± 1517)	0.169
Tumor location:		51 (68.9%)	23 (76.7%)	0.011*
Colon	74 (71.1%)	23 (31.1%)	7 (23.3%)	
Rectum	30 (28.8%)			
Operative procedures:				0.325
Right hemicolectomy	20 (19.2%)	16 (21.6%)	4 (13.3%)	
Extended right hemicolectomy	5 (4.8%)	5 (6.8%)	–	
Left hemicolectomy	6 (5.8%)	5 (6.8%)	1 (3.3%)	
Extended left hemicolectomy	8 (7.7%)	5 (6.8%)	3 (10%)	
Anterior resection	14 (13.5%)	9 (12.2%)	5 (16.7%)	
Low anterior resection	39 (37.5%)	24 (32.4%)	15 (50%)	
Abdominoperineal resection	6 (5.8%)	6 (8.1%)	–	
Total colectomy	6 (5.8%)	4 (5.4%)	2 (6.7%)	
pTNM staging:				0.036*
I	19 (18.3%)	16 (21.6%)	3 (10%)	
IIa	35 (33.7%)	23 (31.1%)	12 (40%)	
IIb	9 (8.7%)	8 (10.8%)	1 (3.3%)	
IIc	1 (1%)	–	1 (3.3%)	
IIIa	5 (4.8%)	3 (4.1%)	2 (6.7%)	
IIIb	26 (25%)	21 (28.4%)	5 (16.7%)	
IIIc	7 (6.7%)	2 (2.7%)	5 (16.7%)	
IVc	1 (1%)	–	1 (3.3%)	
Pathological grading:				0.693
G1 (well differentiated)	22 (21.8%)	17 (23.6%)	5 (17.2%)	
G2 (moderately differentiated)	74 73.3%)	52 (72.2%)	22 (75.9%)	
G3 (poorly differentiated)	5 (5%)	3 (4.2%)	2 (6.9%)	

SD, standard deviation; ASA, American Society of Anesthesiologists; NRS, Nutritional Risk Score-2002; CEA, carcinoembryonic antigen; WBC, White blood cells; pTNM, pathological tumor-node-metastasis

^*^ Variables with p value < 0.05

Vitamin D levels were found to be 15.7 ± 8.3 (13.7; 4.5–60.1) ng/ml in males and 16.4 ± 10.3 (13.5; 5–54.7) ng/ml in females (U = 1237.500, p = 0.776). There was no significant association between 25-OH vitamin D levels and serum CEA, albumin, hemoglobin, and WBC levels. Most of the patients scored ASA-2 (53.8%); patients with higher vitamin D levels were found to have higher ASA scores (p = 0.043).

However, 25-OH vitamin D levels were measured as 18.9 ± 7.4 (20.9; 5–30.4) ng/ml in ASA-1 patients, 15.3 ± 8.7(13.2;4.5–54.7) ng/ml in ASA-2 patients, and 15.5 ± 10.5 (13.15; 5.6–60.1) in ASA-3 patients (x^2^ = 4.485, p = 0.106).

Tumor location was demonstrated as a colon in 74 patients (71.2%) and rectum in 30 patients (28.8%). Metachronous colon tumor was found in 1 patient and synchronous colon tumors in 2 patients. Serum 25-OH vitamin D levels were found to be significantly lower in patients with rectal cancer (13 ± 7.5 (10.3; 4.5–31.7) ng/ml) compared to patients with colon cancer (17.1 ± 9.5 (15.2; 5–60.1) ng/ml) (U = 755.000, p = 0.011). The most common operative procedure performed was low anterior resection (37.5%) followed by right hemicolectomy (19.2%). The laparoscopic approach was chosen in 9 patients, and laparotomy was performed in 95 patients. There were no significant differences observed in vitamin D levels according to the type of operative procedures.

Patients' histopathological results confirmed the diagnosis of carcinoma-in-situ in 2 patients, adenocarcinoma in 88 patients, mucinous adenocarcinoma in 12 patients, signet cell carcinoma in 2 patients, and malignant fibrous histiocytoma in 1 patient. Most of the patients were staged as pTNM stage IIa (33.7%) and IIIb (25%), and a statistically significant difference was found in vitamin D levels for pTNM staging (p = 0.036).

Infectious complications were seen in 18 patients (17.3%), including wound infection, anastomotic leak, intra-abdominal abscess, and pneumonia (Table 2). The anastomotic leak occurred in 7 patients (6.73%): on postoperative day-5 in 1 patient, day-6 in 4 patients, and day-8 in 2 patients. Non-operative management was chosen in 2 patients, and the Hartmann procedure was performed in 5 patients. No significant differences in vitamin D levels were observed according to Clavien-Dindo scores (p = 0.75).

**Table 2:** Short-term outcomes (n = 104)

Variables	N (%) or median (min–max)
Number of patients with complications	25 (24%)
Infectious complications	
Wound Infection	9 (8.6%)
Anastomotic leak	7 (6.73%)
Intra-abdominal abscess	1 (0.96%)
Pneumonia	4 (3.84%)
Non-infectious complications	
Wound dehiscence	3 (2.88%)
Postoperative Ileus	3 (2.88%)
Pneumothorax	1 (0.96%)
Myocardial Infarction	1 (0.96%)
Arrhythmia	1 (0.96%)
Clavien-Dindo Scale	
0	79 (75.9%)
1–2	15 (14.4%)
3–4	8 (7.69%)
5	2 (1.92%)
Length of Hospital Stay (days)	7 (3–29)
30-day mortality	2 (1.92%)

The study population was evaluated for the association between 25-OH vitamin D levels and infectious complications (Table 3). In patients with surgical site infection and infectious complications, 25-OH vitamin D levels were significantly lower than patients without complications (p = 0.036 and p = 0.026). However, no significant difference was demonstrated in 25-OH vitamin D levels according to overall PoC.

**Table 3:** Vitamin D levels for surgical site infection, infectious complications, and PoC are shown

Variables	Study population (n = 104) N (%)	25-OH vitamin D levels** Mean values ± SD (median; min–max) ng/ml	U, p-value
Surgical site infection/yes	9 (8.7%)	16.4 ± 9.1 (14.3; 5–60.1)	U = 246.000, p = 0.036*
Surgical site infection/no	95 (91.3%)	11.4 ± 7.7 (8.3; 4.5–25.9)	
Infectious complications/yes	18 (17.3%)	16.8 ± 9.4 (14.8; 5–60.1)	U = 515.000, p = 0.026*
Infectious complications/no	86 (82.7%)	12.1 ± 6.2 (11.1; 4.5–25.9)	
Postoperative complications/yes	25 (24%)	16.8 ± 9.7 (14.6; 5–60.1)	U = 757.000, p = 0.08
Postoperative complications/no	79 (76%)	13.1 ± 6.2 (11.9; 4.5–25.9)	

^*^ Variables with p value < 0.05

^**^In this hypothesis testing table, the power analysis was 75.3%. To obtain a significant difference between categorical variables of vitamin D levels and infectious complications, the number needed to be treated was 6789. To that end, we chose to use scale variables for vitamin D levels to demonstrate the relation with infectious complications. Since it gives the reason of using vitamin D levels as scale variables while analysing its relation to infectious complications (which is the main outcome of the study), not as subgroups of vitamin D levels

Logistic regression analysis was conducted to evaluate the predictive competence of the preoperative factors for infectious complications (Table 4). Although lower vitamin D levels were associated with infectious complications on univariate analysis, the exact correlation was not shown on multivariate analysis. The type of operative procedure was found to be the only independent risk factor for infectious complications.

**Table 4:** Logistic regression analysis to demonstrate independent risk factors related to infectious complications

Variables	OR (95% CI)	p-value
Gender	4.06 (0.76–21.68)	0.10
Age (years)	1.06 (0.97–1.16)	0.156
NRS	3.8 (0.76–19.01)	0.103
Albumin (gr/dl)	0.41 (0.09–1.78)	0.24
CEA (µg/l)	0.89 (0.76–1.04)	0.14
25-OH vitamin D (ng/ml)	0.92 (0.83–1.02)	0.13
Operative procedure	1.62 (1.08–2.43)	0.019*

CI, confidence interval; NRS, Nutritional Risk Score-2002; CEA, carcinoembryonic antigen

^*^Variables with p value < 0.05

## Discussion

PoC related to colorectal cancer surgery remain a significant problem; infectious complications represent the most of those and have been associated with decreased long-term survival in colorectal cancer patients [24, 25]. Therefore, determining the modifiable risk factors for preoperative complications is particularly significant in patients undergoing colorectal surgery.

In recent years, an increasing number of studies have investigated the effect of 25-OH vitamin D on progression-free survival and overall survival in patients with colorectal cancer [26–28]. It has also been revealed that infectious complications are increased in hospitalized patients with vitamin D deficiency because of its decreased immunomodulatory effects [29, 30]. The effects of vitamin D on the immune system are mainly based on the presence of vitamin D receptors on B and T lymphocytes and macrophages, and promoting chemotaxis, autophagy, and phagolysosomal function in innate immune cells. Vitamin D is also found to be responsible for the barrier function of the intestinal epithelium and the modulation of the bowel system [31].

In line with these findings, preoperative 25-OH vitamin D levels were inversely related to surgical site infections in patients undergoing cardiac surgery and total knee arthroplasty [32, 33]. Quraishi et al. have also reported that hospital-acquired infections are associated with low preoperative 25-OH vitamin D levels in patients following bariatric surgery [34].

To better demonstrate the relation between vitamin D deficiency and PoC, we excluded the patients who were operated on in an emergency setting and received neoadjuvant chemoradiotherapy. To that end, the study cohort was relatively small, consisting of 104 patients with a mean 25-OH vitamin D value of 15.95 (± 9.08) ng/ml. In line with previous studies' findings, patients with surgical site infection and infectious complications had significantly lower 25-OH vitamin D levels compared to patients without complications. However, we did not observe any association between vitamin D levels and overall PoC.

A recent study conducted in hepatobiliary surgery patients revealed that preoperative 25-OH vitamin D levels were related to hospital-acquired infections and in-hospital outcomes [35]. There were also significant differences observed in vitamin D levels according to gender, ASA, and Clavien-Dindo scores. However, there was no significant association between vitamin D levels, gender, ASA, and Clavien-Dindo scores in our study. We suggest that these findings result from the sample size and study cohort, which has a high prevalence (94%) of vitamin D deficiency.

Our secondary outcome was to evaluate the effect of 25-OH vitamin D levels on histopathological grading and pTNM staging based on the fact that decreased 25-OH vitamin D levels are linked with poor survival in colorectal cancer patients [36]. Vitamin D levels were not related to histopathological grading in our study. Värynen et al. revealed that 25-OH vitamin D levels were lower in pTNM stage II-IV colorectal cancer patients than pTNM stage I colorectal cancer patients [16]. Similar to their results, we observed a significant difference in pTNM staging according to vitamin D levels. Moreover, patients with rectal cancer were found to have significantly lower vitamin D levels in the present study. Rectal cancer has also been associated with an increased risk of PoC [24]. Our findings may also suggest a possible relationship between vitamin D levels and PoC.

Abdehgah et al. reported that preoperative 25-OH vitamin D levels were found to be independent predictors of surgical site infections in addition to the length of hospital stay [37]. On multivariate analysis, we could not demonstrate the effect of vitamin D on infectious complications; the only independent risk factor was the type of operative procedure. It should be emphasized that Abdehgah et al. conducted their study in a heterogeneous cohort undergoing varied types of gastrointestinal tract, hepatobiliary, thoracic, and vascular operations. Also, the prevalence of vitamin D deficiency was only 39% in the study cohort.

The major limitation of this observational study was the retrospective analysis of a small sample size with a significantly high prevalence of vitamin D deficiency (94%). Therefore, we could not draw firm results for associations between vitamin D levels and PoC, and Clavien-Dindo scores. Furthermore, dose–response analysis of vitamin D levels for infectious complications could not be performed in this study.

## Conclusions

In summary, vitamin D levels might be a potential risk factor for infectious complications in patients undergoing colorectal cancer surgery. We need prospective randomized controlled studies to draw firm conclusions for the relation of preoperative 25-OH vitamin D levels and PoC and to investigate whether vitamin D levels should be optimized to improve short-term outcomes.

## Data Availability

The datasets used and/or analyzed during the current study are available from the corresponding author on reasonable request.
